# Screening Potential Drugs for COVID-19 Based on Bound Nuclear Norm Regularization

**DOI:** 10.3389/fgene.2021.749256

**Published:** 2021-10-07

**Authors:** Juanjuan Wang, Chang Wang, Ling Shen, Liqian Zhou, Lihong Peng

**Affiliations:** ^1^School of Computer Science, Hunan University of Technology, Zhuzhou, China; ^2^College of Life Sciences and Chemistry, Hunan University of Technology, Zhuzhou, China

**Keywords:** SARS-CoV-2, bounded nuclear norm regularization, virus-drug association, FDA-approved drugs, molecular docking

## Abstract

The novel coronavirus pneumonia COVID-19 infected by SARS-CoV-2 has attracted worldwide attention. It is urgent to find effective therapeutic strategies for stopping COVID-19. In this study, a Bounded Nuclear Norm Regularization (BNNR) method is developed to predict anti-SARS-CoV-2 drug candidates. First, three virus-drug association datasets are compiled. Second, a heterogeneous virus-drug network is constructed. Third, complete genomic sequences and Gaussian association profiles are integrated to compute virus similarities; chemical structures and Gaussian association profiles are integrated to calculate drug similarities. Fourth, a BNNR model based on kernel similarity (VDA-GBNNR) is proposed to predict possible anti-SARS-CoV-2 drugs. VDA-GBNNR is compared with four existing advanced methods under fivefold cross-validation. The results show that VDA-GBNNR computes better AUCs of 0.8965, 0.8562, and 0.8803 on the three datasets, respectively. There are 6 anti-SARS-CoV-2 drugs overlapping in any two datasets, that is, remdesivir, favipiravir, ribavirin, mycophenolic acid, niclosamide, and mizoribine. Molecular dockings are conducted for the 6 small molecules and the junction of SARS-CoV-2 spike protein and human angiotensin-converting enzyme 2. In particular, niclosamide and mizoribine show higher binding energy of −8.06 and −7.06 kcal/mol with the junction, respectively. G496 and K353 may be potential key residues between anti-SARS-CoV-2 drugs and the interface junction. We hope that the predicted results can contribute to the treatment of COVID-19.

## Introduction

Novel coronavirus pneumonia COVID-19, erupted in Wuhan, Hubei, China, has become a global public health challenge (Nittari et al., 2020). By July 26, 2021, it has caused 192,284,207 confirmed cases and 4,128,152 deaths (WHO, 2021). Although the COVID-19 vaccine has been researched and developed in many countries and regions, it still fails to avoid the risk of infection. Therefore, it is an urgent task to design effective drugs for the COVID-19 treatment (Khan et al., 2020a).

COVID-19 is caused by SARS-CoV-2 infection. SARS-CoV-2, like most coronaviruses, is a positive single stranded virus with unique coronal protein spikes (Khan et al., 2020b). It invades human body through SARS-CoV-2 Spike (S) protein binding with the surface of host angiotensin-converting enzyme 2 (ACE2) (Morse et al., 2020). Based on the homology between SARS-CoV-2 and other RNA viruses (such as SARS-CoV and MERS-CoV), we can investigate RNA virus-related FDA-approved drugs to find possible chemical agents for preventing COVID-19.

Computational methods for identifying potential antiviral drugs against COVID-19 contain structure-based methods and network-based methods. Structure-based methods are a pivotal implement based on computer-aided drug design and structural molecular biology. The type of methods aims at predicting binding sites between chemical agents and target proteins and thus elucidating basic biochemical processes (McConkey et al., 2002). Lan et al. (2020) determined the crystal structure of receptor-binding domain (RBD) in which the S protein binds to ACE2. Li et al. (2021) screened 21 antiviral, antifungal and anticancer compounds to identify possible SARS-CoV-2 papain inhibitors based on silicon molecular docking. Elfiky, 2020a utilized sequence analysis and molecular docking to construct an anti-COVID-19 RNA-dependent RNA polymerase (RdRp) prediction model. Panda et al. (2020) used molecular docking technique to implement virtual screening among SARS-CoV-2 protein, main protease, and RDB/ACE2 complex and FDA-approved antiviral drugs. Maurya et al. (2020) screened possible antiviral natural products against the S protein and its cellular receptor from Ayurveda through molecular docking. Choudhary et al. (2020) utilized a structure-based virtual screening technique to find possible inhibitors for SARS-CoV-2 entering cells. Wang et al. (2020a) investigated the development of structure-based methods and emphasized the limitations and further works of anti-SARS-CoV-2 drug research. Gahlawat et al. (2020) exploited structure-based virtual screening technique to investigate the inhibition effects of major proteases in coronavirus.

Network-based methods have been broadly applied to anti-SARS-CoV-2 drug screening. For example, Peng et al. (2020) built one virus-drug (VDA) association dataset and employed a regularized least square classifier to explore the therapeutic clues of COVID-19 by combining drug chemical structures, virus complete genome sequences, bipartite local model and neighborhood association information. Zhou L. et al. (2020) exploited a KATZ algorithm (VDA-KATZ) to predict candidate drugs for the SARS-CoV-2 prevention on the VDA dataset. Peng et al. (2021) continued to construct two VDA datasets and developed a random walk with restart method (VDA-RWR) to prioritize drugs related to COVID-19. Zhou Y. et al. (2020) designed a formidable network-based method to reposition the existing chemical agents and quickly screened latent drug combinations for COVID-19. Taz et al. (2021) identified the infectious responses between SARS-CoV-2 and idiopathic pulmonary fibrosis-infected lung cells based on protein-protein interaction network. Du et al. (2021) probed a network-based virus-host interaction prediction method and considered its application on SARS-CoV-2. Messina et al. (2020) studied pathogenesis of SARS-CoV-2 infection to discover the etiopathogenesis of COVID-19 by analyzing virus-host interactome. Ahmed (2020) found that Vitamin D may inhibit SARS-CoV-2 infection based on a network analysis approach. Stolfi et al. (2020) used a broader molecular map to reveal potential therapeutic strategy for COVID-19.

Computational methods effectively prioritize potential drugs for the SARS-CoV-2 infection. In this work, we propose a Virus-Drug Association (VDA) prediction algorithm, VDA-GBNNR, to discover potential chemical agents against COVID-19 based on virus similarity, drug similarity, VDA network, Gaussian Association Profile Kernel (GAPK), and Bounded Nuclear Norm Regularization (BNNR). VDA-GBNNR is compared with three existing VDA prediction methods, that is, VDA- RLSBN (Peng et al., 2020), VDA-KATZ (Zhou L. et al., 2020), VDA-RWR (Peng et al., 2021) and one network-based microRNA-anticancer drug association prediction model SMiR-NBI (Li et al., 2016) on three VDA datasets. The experimental results show that VDA-GBNNR computes the best AUC, accuracy, sensitivity, and specificity. In addition, the inferred top six antiviral drugs against SARS-CoV-2, remdesivir, favipiravir, ribavirin, mycophenolic acid, niclosamide, and mizoribine come together in any two datasets. Molecular dockings between the six compounds and the junction of SARS-CoV-2 S protein and human ACE2 are implemented to calculate molecular binding energies and identify binding sites between them. Niclosamide and mizoribine are found to have the strongest binding energy of −8.06 and −7.06 kcal/mol with the junction, respectively.

## Materials and Methods

**FIGURE 1:**
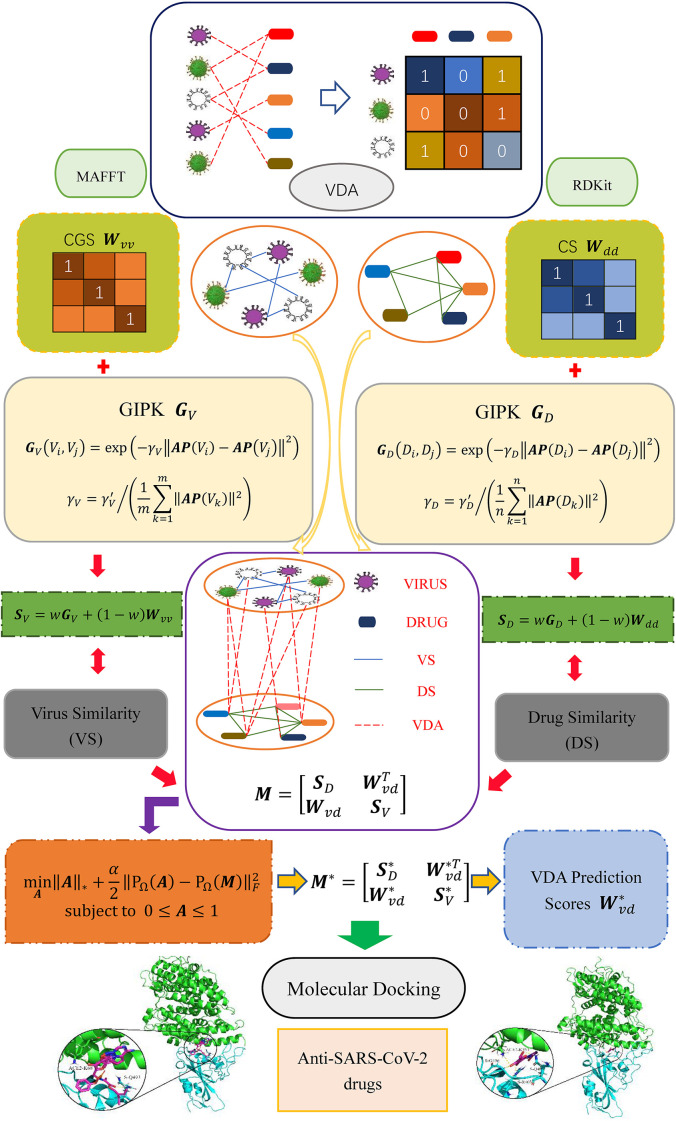
Overall flow chart of VDA-GBNNR.

In this study, inspired by the works provided by Chen and Huang (2017); Chen et al. (2018b), Yang et al. (2019), and Liu et al. (2020) we develop a VDA prediction framework (VDA-GBNNR) to screen underlying drugs for inhibiting COVID-19. First, virus similarity and drug similarity are calculated based on virus complete genomic sequences, drug chemical structures, and Gaussian Association Profiles (AP). Second, a BNNR model is developed to complete unknown associations between viruses and drugs. Finally, the predicted top anti-SARS-CoV-2 drugs are docked with the junction of the S protein bound with ACE2. The overall workflow is shown in Figure 1.

### Datasets

Three VDA datasets are obtained from Peng et al. (2021). Each dataset contains virus similarity matrix, drug similarity matrix, and VDA matrix. In each dataset, virus complete genomic sequences were downloaded from the NCBI database (Coordinators, 2018), and MAFFT (Katoh et al., 2019) (a multi-sequence alignment tool) was utilized to compute virus sequence similarity matrix **W**_*vv*_. Drug chemical structures were obtained from DrugBank (Wishart et al., 2018) and RDKit (an open-source chemical information software) was used to calculate drug chemical structure similarity matrix **W**_*dd*_. VDA matrix **W**_*vd*_ is achieved by searching the DrugBank, NCBI, and PubMed (Motschall and Falck-Ytter, 2005) databases. In **W**_*vd*_, *W*_*ij*_ = 1 if virus *v*_*i*_ interacts with drug *d*_*j*_; otherwise, *W*_*ij*_ = 0. Table 1 shows the details of three VDA datasets.

**TABLE 1:** Details of three VDA datasets.

Datasets	Dataset 1	Dataset 2	Dataset 3
Number of viruses (*m*)	12	69	34
Number of drugs (*n*)	78	128	203
Number of VDAs	96	770	407
Proportion of VDA to all virus-drug pairs	10.26%	8.72%	5.90%

### Similarity Computation

#### GAPK Similarity

For a given virus *v*_*i*_, the Gaussian association profile **AP**(*v*_*i*_) is defined as the *i*th row of a VDA matrix **W**_*vd*_ to describe its association information with all drugs. GAPK similarity between two viruses [i.e., (*v*_*i*_, *v*_*j*_)] is calculated by Eq. (1).


(1) $$\begin{array}{c} {\text{G}}_{V}\,\left(v_{i},v_{j}\right)=\exp\left(-\gamma_{v}\,{||\text{AP}\,\left(v_{i}\right)-\text{AP}\,\left(v_{j}\right)||}^{2}\right)\\ \gamma_{v}=\gamma_{v}^{\prime }/\left(\frac{1}{m}\,\sum_{k=1}^{m}{||\text{AP}\,\left(v_{k}\right)||}^{2}\right) \end{array}$$

where γ_*v*_ represents normalized kernel bandwidth based on bandwidth parameter $\gamma_{v}^{\prime }$, and *m* is the number of viruses.

For a given drug *d*_*i*_, its Gaussian association profile **AP**(*d*_*i*_) is defined as the *i*th column of a VDA matrix **W**_*vd*_ to describe its association information with all viruses. GAPK similarity between two drugs [i.e., (*d*_*i*_, *d*_*j*_)] is computed by Eq. (2):


(2) $$\begin{array}{c} {\text{G}}_{D}\,\left(d_{i},d_{j}\right)=\exp\left(-\gamma_{d}\,{||\text{AP}\,\left(d_{i}\right)-\text{AP}\,\left(d_{j}\right)||}^{2}\right)\\ \gamma_{d}=\gamma_{d}^{\prime }/\left(\frac{1}{n}\,\sum_{k=1}^{n}{||\text{AP}\,\left(d_{k}\right)||}^{2}\right) \end{array}$$

where γ_*d*_ indicates normalized kernel bandwidth based on bandwidth parameter $\gamma_{d}^{\prime }$, and *n* is the number of drugs.

#### Similarity Integration

Complete genomic sequence similarity **W**_*vv*_, chemical structure similarity **W**_*dd*_, and GAPK similarity (**G**_*V*_ and **G**_*D*_) are integrated to compute the final virus similarity matrix **S**_*V*_ (Eq. 3) and drug similarity **S**_*D*_ (Eq. 4). The parameter *w* is introduced to measure the importance between biological similarity and GAPK similarity.


(3) $${\text{S}}_{V}=w\,{\text{G}}_{V}+\left(1-w\right)\,{\text{W}}_{v\,v}$$

(4) $${\text{S}}_{D}=w\,{\text{G}}_{D}+\left(1-w\right)\,{\text{W}}_{d\,d}$$

### Heterogeneous Network Construction

A heterogeneous virus-drug network is constructed by integrating virus similarity network, drug similarity network and VDA network. The edge between two viruses/drugs is weighted according to their similarity. The heterogeneous network can be represented as a bipartite graph *G*(**V**, **D**, **E**), where **E**(*G*) = {*e*_*ij*_} ⊆ **V** × **D**, *e*_*ij*_ represents the edge between the virus *v*_*i*_ and the drug *d*_*j*_, **V** and **D** represent virus set and drug set, respectively. The adjacency matrix of the heterogeneous network is defined as Eq. (5).


(5) $$\text{M}=\left[\begin{array}{cc} {\text{M}}_{d\,d} & {\text{W}}_{v\,d}^{T}\\ {\text{W}}_{v\,d} & {\text{M}}_{v\,v} \end{array}\right]$$

where **W**_*vd*_ denotes known VDA matrix, **M**_*dd*_ and **M**_*vv*_ represent the adjacency matrices of drug similarity network and virus similarity network, respectively. Hence, the adjacency matrix can be rewritten as Eq. (6).


(6) $$\text{M}=\left[\begin{array}{cc} {\text{S}}_{D} & {\text{W}}_{v\,d}^{T}\\ {\text{W}}_{v\,d} & {\text{S}}_{V} \end{array}\right]$$

### VDA-GBNNR Model

In three VDA datasets, known VDAs in the matrix **W**_*vd*_ account for about 10.26, 8.72, and 5.90% among all possible virus-drug pairs, respectively. That is, the majority of virus-drug pairs are unlabeled and need to be completed. Therefore, we aim to complete unknown elements through a bounded nuclear norm regularization model.

The rank of a matrix describes information redundancy, and lower rank denotes less information redundance. Indeed, VDA prediction can be represented as a low-rank matrix completion problem. Therefore, we built the following model to complete the missing association information in a VDA matrix by Eq. (7):


(7) minrA⁢a⁢n⁢k⁢(A)subject⁢to⁢PΩ⁢(A)=PΩ⁢(M)

where **A** is a matrix after completion, *rank*(⋅) indicates the rank of a matrix, **M** ∈ ℛ ^(*m* + *n*) × (*m* + *n*)^ is a given VDA matrix, Ω is the set of index pairs (*i, j*) which contains all known VDAs in **M**, and P_Ω_ is the projection operator on Ω.


(8) (PΩ⁢(A))i⁢j={Ai⁢j,(i,j)∈Ω 0,(i,j)∉Ω

The solution of *rank* (**A**) in Eq. (7) is a non-convex problem. Based on the nuclear norm optimization provided by Candes and Recht (2013), the model Eq. (7) can be solved by Eq. (9):


(9) $$\begin{array}{c} \min_{A}{||A||}_{*}\\ \text{subject}\,{\text{toP}}_{\Omega }\,\left(A\right)={\text{P}}_{\Omega }\,\left(M\right) \end{array}$$

where ||**A**||_*_ denotes the nuclear norm of **A** and can be obtained by summating all singular values in *A*.

The elements in virus similarity matrix **W**_*vv*_ and drug similarity matrix **W**_*dd*_ are between 0 and 1, and the elements in VDA matrix **W**_*vd*_ are either 1 or 0. Therefore, the predicted association scores for unknown virus-drug pairs are expected to be between 0 and 1. The value closer to 1 denotes bigger probability that a virus and a drug pair is linked, and vice versa. However, the elements in Eq. (9) may be any real value in (−∞, + ∞). To ensure that the predicted results are within the interval of [0, 1], a bounded constraint is added to Eq. (9). In addition, there may exist data noise when evaluating virus similarities and drug similarities. To solve this problem, we build a matrix completion model with noise tolerance based on rank minimization by Eq. (10):


(10) $$\begin{array}{c} \min_{A}{||A||}_{*}\\ \text{subject}\,\text{to}\,{||{\text{P}}_{\Omega }\,\left(A\right)-{\text{P}}_{\Omega }\,\left(M\right)||}_{F}\leq \in \end{array}$$

where ||⋅||_*F*_ denotes Frobenius norm and ∈ indicates the noise level.

Since the noise level is unknown, it is difficult to choose the most appropriate parameters in Eq. (10). Therefore, a soft regularization term is introduced to tolerate unknown noise and reduce computational complexity. Thus, a bound nuclear norm regularization model (VDA-GBNNR) is developed to screen possible associations between viruses and drugs by Eq. (11):


(11) minA||A||*+α2⁢||PΩ⁢(A)-PΩ⁢(M)||F2subject⁢to⁢ 0≤A≤1

where α is a parameter used to balance the nuclear norm and the error term, and each element in **A** satisfies 0 ≤ *A*_*ij*_ ≤ 1.

Through introducing an auxiliary matrix **W**, Eq. (11) can be optimized using alternating direction method of multipliers defined by Eq. (12).


(12) $$\begin{array}{c} \min_{A}{||A||}_{*}+\frac{\alpha }{2}\,{||{\text{P}}_{\Omega }\,\left(W\right)-{\text{P}}_{\Omega }\,\left(M\right)||}_{F}^{2}\\ \text{subject}\,\text{to}\,\text{A}=\text{W}\\ 0\leq \text{W}\leq 1 \end{array}$$

where the initial term **A**_1_ = P_Ω_ (**M**). Consequently, the augmented Lagrange function can be defined by Eq. (13).


$$L\,\left(W,A,B,\alpha ,\beta \right)={||A||}_{*}+\frac{\alpha }{2}\,{||{\text{P}}_{\Omega }\,\left(W\right)-{\text{P}}_{\Omega }\,\left(M\right)||}_{F}^{2}+T_{r}$$

(13) $$\left({\text{B}}^{T}\,\left(\text{A}-\text{W}\right)\right)+\frac{\beta }{2}\,{||\text{A}-\text{W}||}_{F}^{2}$$

where **B** denotes the Lagrange multiplier and β > 0 indicates the penalty parameter. At each iteration, VDA-GBNNR alternatively calculates **W**_*k* + 1_, **A**_*k* + 1_ and **B**_*k* + 1_ by fixing other two terms. The specific solutions about **W**_*k* + 1_, **A**_*k* + 1_ and **B**_*k* + 1_ were provided by Yang et al. (2019). VDA-GBNNR can update VDA matrix ${\text{W}}_{v\,d}^{*}$ by completing the missing elements in *W*_*vd*_.

## Results

### Evaluation Metrics

In this study, sensitivity, specificity, accuracy, and AUC are used to evaluate the performance of our proposed VDA-GBNNR method. Accuracy denotes the proportion of correctly inferred positive and negative VDAs to all positive and negative VDAs. Sensitivity denotes the ratio of correctly predicted positive VDAs to all positive VDAs. Specificity represents the rate of correctly identified negative VDAs to all negative VDAs. The details are defined by Eqs. (14)–(16):


(14) $$A\,c\,c\,u\,r\,a\,c\,y=\frac{T\,P+T\,N}{T\,P+T\,N+F\,P+F\,N}$$

(15) $$S\,e\,n\,s\,i\,t\,i\,v\,i\,t\,y=\frac{T\,P}{T\,P+F\,N}$$

(16) $$S\,p\,e\,c\,i\,f\,i\,c\,i\,t\,y=\frac{T\,N}{T\,N+F\,P}$$

where TP, FP, FN, and TN indicate true positive, false positive, false negative, and true negative, respectively.

AUC denotes Area Under the Receiver Operating Characteristic (ROC) Curve. In the curve, the horizontal axis indicates False Positive Rate (FPR) and the vertical axis indicates True Positive Rate (TPR). FPR denotes the proportion of predicted false positive VDAs to all negative VDAs and TPR demonstrates the proportion of true positive VDAs to all positive VDAs. They are defined by Eqs. (17)–(18):


(17) $$T\,P\,R=\frac{T\,P}{\left(T\,P+F\,N\right)}$$

(18) $$F\,P\,R=\frac{F\,P}{\left(F\,P+T\,N\right)}$$

### Experimental Settings and Parameter Selection

In the experiment, we conduct fivefold cross validation for 10 times to evaluate the performance of VDA-GBNNR. Eighty percent of elements in the VDA matrix **W**_*vd*_ are randomly selected as the training set and the remaining is used the testing set. Parameters α, β, *w*, and γ′ are set in the range of [0.1, 1, 10, 100], [0.1, 1, 10, 100], [0, 0.1, 0.2,…, 1], and [0.5, 1, 1.5,…, 3], respectively. The optimal parameter combination is obtained by grid search. Table 2 shows parameter combinations when the top 10 AUCs are confirmed based fivefold cross validation for 10 times.

**TABLE 2:** Parameter settings for the top 10 AUCs.

Rank	Dataset 1					Dataset 2					Dataset 3				
	α	β	w	γ′	AUC	α	β	w	γ′	AUC	α	β	w	γ′	AUC
1	1	0.1	0.5	0.5	0.9013	1	10	0.4	0.5	0.8564	1	10	0.2	0.5	0.8818
2	1	1	0.5	0.5	0.9010	1	10	0.3	0.5	0.8562	1	1	0.7	0.5	0.8804
3	1	1	0.5	1	0.9002	1	10	0.2	0.5	0.8559	1	10	0.1	0.5	0.8799
4	1	1	0.4	0.5	0.8994	1	10	0.5	1	0.8557	1	10	0.1	1	0.8795
5	1	0.1	0.4	0.5	0.8977	1	10	0.3	1	0.8554	1	1	0.6	0.5	0.8794
6	1	0.1	0.5	1	0.8919	1	10	0.4	1	0.8553	1	10	0.3	0.5	0.8792
7	1	0.1	0.4	1	0.8907	10	10	0.4	1	0.855	1	10	0	2.5	0.8782
8	1	1	0.4	1	0.8896	10	10	0.5	1	0.8546	1	10	0	2	0.8782
9	1	1	0.3	0.5	0.8894	1	10	0.5	0.5	0.8544	1	10	0	1.5	0.8775
10	1	1	0.6	1	0.8878	1	10	0.4	1.5	0.854	1	0.1	0.5	0.5	0.8775

Table 3 shows the optimal parameter settings for VDA-KATZ, VDA-RLSBN, VDA-RWR, and VDA-GBNNR based on grid search. The four methods obtain the best performance when parameters are set the corresponding values provided by Table 3. In the SMiR-NBI method, there is no parameter to set.

**TABLE 3:** Optimal parameter settings for different models.

	VDA-KATZ	VDA-RLSBN	VDA-RWR	VDA-GBNNR
Dataset 1	β = 0.04 *w*_1_ = *w*_2_ = 0.9 $\gamma_{v}^{\prime }=\gamma_{d}^{\prime }=2.5$	α = 0.4	*r* = 0.7 μ = 0.9 α = 0.5	α = 1 β = 0.1 *w* = 0.5 γ′ = 0.5

Dataset 2	β = 0.06 *w*_1_ = *w*_2_ = 0.3 $\gamma_{v}^{\prime }=\gamma_{d}^{\prime }=1.0$	α = 0.1	*r* = 0.7 μ = 0.9 α = 0.5	α = 1 β = 10 *w* = 0.3 γ′ = 0.5

Dataset 3	β = 0.05 *w*_1_ = *w*_2_ = 0.7 $\gamma_{v}^{\prime }=\gamma_{d}^{\prime }=2.5$	α = 0.1	*r* = 0.7 μ = 0.9 α = 0.5	α = 1 β = 10 *w* = 0.1 γ′ = 0.5

### Performance Comparison With Other Methods

To evaluate the performance of VDA-GBNNR, we compare it with four classical association prediction methods based on fivefold cross validation, that is, SMiR-NBI, VDA-RLSBN, VDA-KATZ, and VDA-RWR. SMiR-NBI prioritized miRNAs as possible biomarkers to depict their responses to anticancer drug therapy on a heterogeneous drugs-miRNA network. VDA-KATZ, VDA-RLSBN, and VDA-RWR are the newest three VDA prediction algorithms. The experiments are implemented for 100 times and the average performance is taken as the final results. Table 4 gives sensitivities, specificities, accuracies, and AUCs of the five VDA identification models on the three VDA datasets.

**TABLE 4:** Performance comparison of different methods.

Datasets	Methods	Sensitivity	Specificity	Accuracy	AUC
Dataset 1	SMiR-NBI	0.8342	0.1925	0.2069	0.5728
	VDA-KATZ	0.6976	0.6684	0.6691	0.8803
	VDA-RLSBN	**0.9279**	**0.9841**	**0.9298**	**0.9085**
	VDA-RWR	0.4824	0.7831	0.8278	0.8582
	VDA-GBNNR	0.8224	0.8460	0.8400	0.8965

Dataset 2	SMiR-NBI	0.8339	0.0939	0.1078	0.4146
	VDA-KATZ	0.5512	0.7574	0.7535	0.8296
	VDA-RLSBN	0.5517	0.7391	0.7228	0.7873
	VDA-RWR	0.5022	0.6643	0.6613	0.6675
	VDA-GBNNR	**0.8358**	**0.8425**	**0.8365**	**0.8562**

Dataset 3	SMiR-NBI	**0.9232**	0.0431	0.0540	0.4378
	VDA-KATZ	0.7116	0.5666	0.5684	0.8478
	VDA-RLSBN	0.7004	0.6048	0.6102	0.8264
	VDA-RWR	0.5053	0.7057	0.7032	0.7123
	VDA-GBNNR	0.8611	**0.8519**	**0.8482**	**0.8803**

*The bold values represent the best performance among five VDA prediction methods.*

From Table 4, it can be seen that VDA-RLSBN obtains better performance than VDA-GBNNR in dataset 1. However, VDA-GBNNR achieves the best sensitivity, specificity, accuracy, and AUC on dataset 2 and the best specificity, accuracy, and AUC on dataset 3, significantly outperforming other four VDA prediction methods including VDA-RLSBN. For example, in dataset 2, VDA-GBNNR computes the highest accuracy of 0.8365, better 87.11, 9.92, 13.59, and 20.94% than SMiR-NBI, VDA-KATZ, VDA-RLSBN, and VDA-RWR, respectively. VDA-GBNNR still calculates the best AUC of 0.8562, better 51.58, 3.11, 8.05, and 22.04% than the four methods, respectively.

**FIGURE 2:**
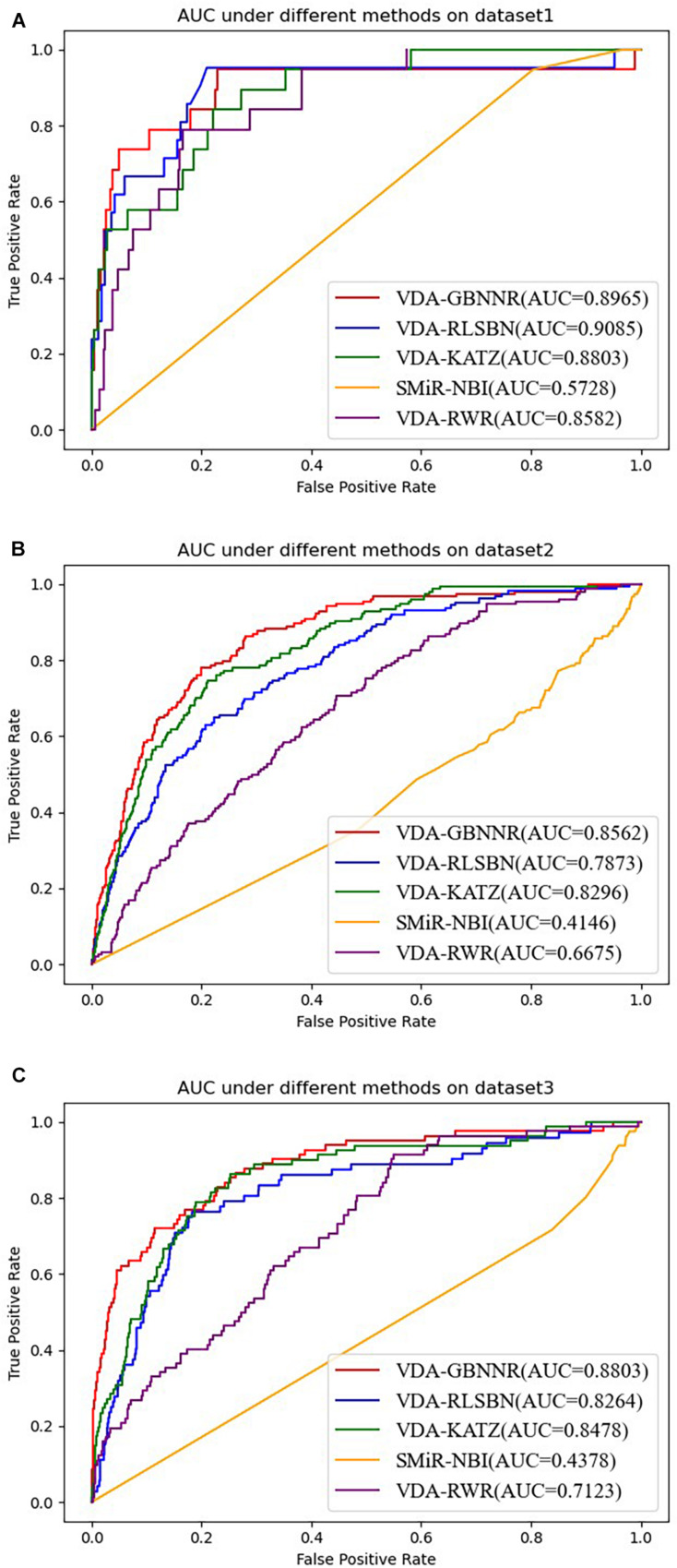
The AUC values of five VDA prediction models on three datasets. **(A)** The AUC values of five VDA prediction models on dataset 1. **(B)** The AUC values of five VDA prediction models on dataset 2. **(C)** The AUC values of five VDA prediction models on dataset 3.

On dataset 3, although SMiR-NBI obtains the best sensitivity of 0.9232, the performance calculated by VDA-GBNNR significantly outperforms SMiR-NBI in terms of specificity, accuracy, and AUC. VDA-GBNNR computes the highest accuracy of 0.8482, better 93.63, 32.99, 28.06, and 17.10% than SMiR-NBI, VDA-KATZ, VDA-RLSBN, and VDA-RWR, respectively. VDA-GBNNR achieves the best AUC of 0.8803, higher 50.27, 3.69, 6.12, and 19.08% than the above four methods, respectively. Figure 2 shows the AUC values computed by five VDA prediction models on three VDA datasets.

### Performance of BNNR With Gaussian Kernel or Not

**FIGURE 3:**
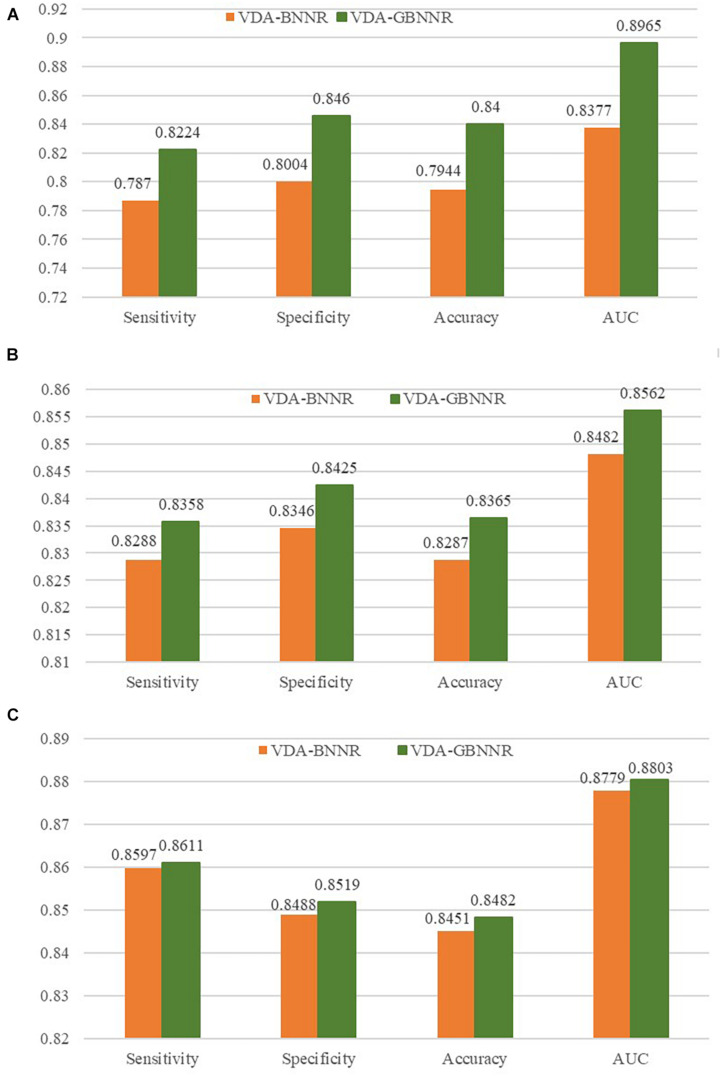
Performance comparison between VDA-BNNR and VDA-GBNNR on three datasets. **(A)** Performance comparison between VDA-BNNR and VDA-GBNNR on dataset 1. **(B)** Performance comparison between VDA-BNNR and VDA-GBNNR on dataset 2. **(C)** Performance comparison between VDA-BNNR and VDA-GBNNR on dataset 3.

In this section, we investigate the effect of GAPK on the prediction performance. In the BNNR model (VDA-BNNR) without GAPK, the adjacent matrix $\text{M}=\left[\begin{array}{cc} {\text{W}}_{d\,d} & {\text{W}}_{v\,d}^{T}\\ {\text{W}}_{v\,d} & {\text{W}}_{v\,v} \end{array}\right]$ is used to represent the heterogeneous virus-drug network where **W**_*vv*_ and **W**_*dd*_ denote virus complete genomic sequence similarity and drug chemical structure similarity, respectively. The comparison results are illustrated in Figure 3. From Figure 3, we can observe that VDA-GBNNR improves the prediction performance compared to VDA-BNNR.

### Case Study

After confirming the prediction performance of VDA-GBNNR, we further discover potential available drugs applied to the treatment of COVID-19. Small molecules with the top 10 association scores with SARS-CoV-2 are shown in Tables 5–7. In addition, we search the recent documents and find that all the inferred top 10 chemical agents have been reported by COVID-19-related publications in the three datasets. In particular, remdesivir, favipiravir, ribavirin, mycophenolic acid, niclosamide and mizoribine come together in any two datasets.

**TABLE 5:** The predicted top 10 antiviral drugs against SARS-CoV-2 in dataset 1.

Number	Drugs	References
1	Remdesivir	PMID: 33857725, 33267759, 32258351, 32127666, 32020029, 32282022, 32023685, 32022370, 32297571, 32035533, 31971553, 31996494
2	Oseltamivir	PMID: 32127666, 32297571, 32034637, DOI: 10.1038/d41573-020-00016-0
3	Zanamivir	PMID: 32294562, 32511320
4	Laninamivir	DOI: 10.3389/fneur.2020.0051
5	Presatovir	PMID: 32147628, 33281124
6	Peramivir	DOI: 10.1101/2020.07.13.20180
7	Valganciclovir	DOI: 10.1002/med.21776
8	Maribavir	DOI: 10.1002/med.21776
9	Mizoribine	PMID: 32886002 DOI: 10.1152/ajpheart.00506.2020.
10	Baloxavir marboxil	PMID: 32127666, 32373347

**TABLE 6:** The predicted top 10 antiviral drugs against SARS-CoV-2 in dataset 2.

Number	Drugs	References
1	Favipiravir	PMID: 32869558, 32282022, 32297571, 33130203, 32127666, 32346491, 32967849, 32972430, 32246834
2	Remdesivir	PMID: 33857725, 33267759, 32258351, 32127666, 32020029, 32282022, 32023685, 32022370, 32297571, 32035533, 31971553, 31996494
3	Ribavirin	PMID: 33550050, 32222463, 32127666, 32869558, 32282022, 33556871, 32034637, 32227493, 26492219, 32771797, DOI: 10.1038/d41573-020-00016-0
4	Mycophenolic acid	PMID: 33525411, 32579258
5	Cidofovir	PMID: 32546018, DOI: 10.1007/s10067-020-05133-0
6	Itraconazole	DOI: 10.22541/au.159467021.16927198
7	Niclosamide	PMID: 32125140, 33689873
8	Pleconaril	DOI: 10.30493/DLS.2020.225404
9	Cyclosporine	PMID: 33385128
10	BCX4430 (Galidesivir)	PMID: 32127666, 32711596

**TABLE 7:** The predicted top 10 antiviral drugs against SARS-CoV-2 in dataset 3.

Number	Drugs	References
1	Ribavirin	PMID: 33550050, 32222463, 32127666, 32869558, 32282022, 33556871, 32034637, 32227493, 26492219, 32771797, DOI: 10.1038/d41573-020-00016-
2	Nitazoxanide	PMID: 32127666, 32568620, 32448490
3	Chloroquine	PMID: 32127666, 32020029, 32023685, 32282022, 32297571, 32145363, 32074550, 32236562
4	Favipiravir	PMID: 32869558, 32282022, 32297571, 33130203, 32127666, 32346491, 32967849, 32972430, 32246834
5	Camostat	PMID: 32347443
6	Niclosamide	PMID: 32125140, 33689873
7	Umifenovir	PMID: 32127666, 32941741
8	Hexachlorophene	PMID: 15950190
9	Mizoribine	PMID: 32886002, DOI: 10.1152/ajpheart.00506.2020.
10	Mycophenolic acid	PMID: 33525411, 32579258

Remdesivir is an intravenous nucleotide prodrug bound with viral RdRp and can inhibit viral replication through premature termination of RNA transcription (Amirian and Levy, 2020). It has been validated to be able to inhibit the replication of SARS-CoV and MERS-CoV (Sheahan et al., 2017). The drug has obtained an emergency use authorization to treat the patients infected by SARS-CoV-2 from the Food and Drug Administration (FDA) (Moirangthem and Surbala, 2020).

Favipiravir is a guanosine analog targeting RdRp and blocking the rhinoviruses replication (Kocayiğit et al., 2021). The drug is effective against a large-scale grippe virus types and subtypes (Furuta et al., 2017). Two recent open-label experiments discovered its therapeutic effective on COVID-19 (Cai et al., 2020; Prakash et al., 2020). It has been also applied to the treatment of COVID-19 by the Japanese government (Hoang and Anh, 2020), and exhibited hopeful results in clinical researches in Russia and China. More importantly, its anti-SARS-CoV-2 experiments are conducting in the United States, the United Kingdom and India (Joshi et al., 2020).

Ribavirin is an antiviral drug against hepatitis C virus and other RNA viruses (Tian et al., 2021). It can inhibit viral RNA synthesis and hander normal viral replication by binding to viral RNA (Kim et al., 2019). It combines closely with RdRp and is a powerful antiviral drug against SARS-CoV-2 (Elfiky, 2020b). Clinical trials about the treatment of ribavirin on the patient with COVID-19 have been conducted (Zarandi et al., 2021).

Mycophenolic acid is an antibiotic extracted from penicillium species. Mycophenolic acid can block the production of guanosine monophosphate by inhibiting inosine monophosphate dehydrogenase. Mycophenolic acid is also an immunosuppressive drug with a strong anti-proliferation effect (Kim et al., 2019). Studies suggest that mycophenolic acid has a potential inhibitory effect on the enzyme reproduced by SARS-CoV-2 (Muhseen et al., 2021).

Niclosamide is an oral bioavailable chlorosalicylanilide with deworming and potential anti-tumor effect (Kim et al., 2019). Niclosamide has various biological activities, for instance, anti-tuberculosis activity (Piccaro et al., 2013), antibacterial activity (Imperi et al., 2013), anticancer activity (Osada et al., 2011), and extensive antiviral activity resistant to coronaviruses (SARS-CoV and MERS-CoV) (Xu et al., 2020). Niclosamide can prevent cells from the cytopathic impact produced by the SARS-CoV-2 infection, suggesting that it may be applied to threat the COVID-19 pandemic (Shamim et al., 2021).

Mizoribine is an imidazole nucleoside antibiotic isolated from bacillus brucellosis (Mizuno et al., 1974). Mizoribine lacks antimicrobial activity, however, it has powerful immunosuppressive activity and has been used in clinic after kidney transplantation (Tajima et al., 1984). It may be used as a potentially beneficial drug for hypertensive patients infected by COVID-19 (Jakovac, 2020).

### Molecular Docking

Inspired by molecular docking provided by Peng et al. (2021), we further investigate the binding energy between the predicted six anti-SARS-CoV-2 drugs and the junction of the S protein-ACE2 interface by molecular docking. The chemical structures of the overlapping six small molecules are achieved from DrugBank in the PDB format. The PDB file is then converted to the PDBQT format by AutoDock4 (Morris et al., 2009). The structure of the S protein bound with ACE2 is downloaded from the RCSB Protein Data Bank (Burley et al., 2017), and the PDBID number is 6M0J. The predicted drugs are then regarded as ligands, and the junction of the S protein-ACE2 interface is regarded as receptors.

Table 8 illustrates molecular docking results including molecular binding energies and binding sites. It can be observed that the six drugs have higher molecular docking energies with the junction of the S protein-ACE2 interface. More importantly, the key residues between the six small molecules and the interface junction are Q493 and K68 for remdesivir, G496 and K353 for favipiravir, G496, Q493, R403, and K353 for ribavirin, G496, F390, and K353 for mycophenolic acid, E37 for niclosamide, and N439, Q506, N330, and Q325 for mizoribine. In addition, the results suggest that G496 and K353 may be potential key residues between anti-SARS-CoV-2 drugs and the interface junction.

**TABLE 8:** Molecular docking between antiviral drugs and the junction of the S protein-ACE2 interface.

Ligands	Molecular formula	Binding energy (kcal/mol)	Binding sites
Remdesivir	*C*_27_ *H*_35_ *N*_6_ *O*_8_ *P*	−7.00	Q493
			K68
Favipiravir	*C*_5_ *H*_4_ *FN*_3_ *O*_2_	−5.32	G496
			K353
Ribavirin	*C*_8_ *H*_12_ *N*_4_ *O*_5_	−6.59	496, Q493, R403
			K353
Mycophenolic acid	*C*_17_ *H*_20_ *O*_6_	−7.00	G496
			F390, K353
Niclosamide	*C*_13_ *H*_8_ *C* *l*_2_ *N*_2_ *O*_4_	−8.06	E37
Mizoribine	*C*_9_ *H*_13_ *N*_3_ *O*_6_	−7.06	N439, Q506
			N330, Q325

**FIGURE 4:**
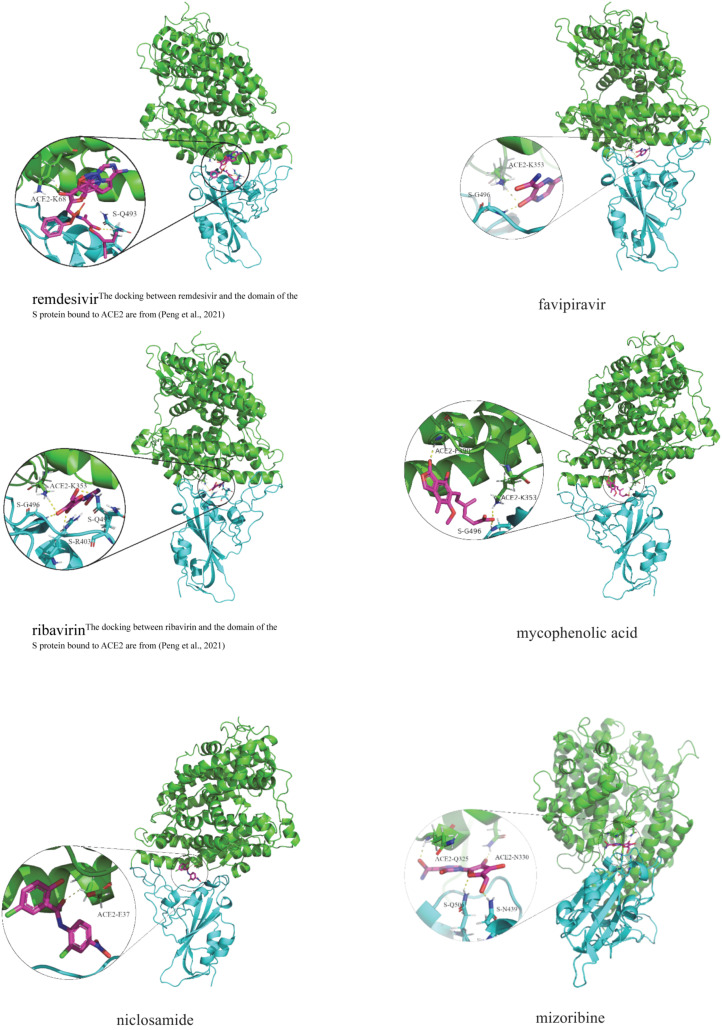
Molecular docking between the predicted six anti-SARS-CoV-2 drugs and the domain of the S protein bound to ACE2.

Figure 4 demonstrates the dockings between remdesivir, favipiravir, ribavirin, mycophenolic acid, niclosamide and mizoribine and the junction of the S protein-ACE2 interface. In the figure, cyan indicates the S protein, green represents human ACE2, and the circles in each subgraph denotes key residues.

## Discussion

With the rapid spread of SARS-CoV-2, it is vital to screen specific drugs for patients infected by COVID-19. Although vaccines have been launched, it is well known that the effect of vaccines for SARS-CoV-2 is mainly prevention, rather than treatment. After vaccination, it cannot completely ensure that people will not be infected by SARS-CoV-2. Therefore, it is an urgent task to find possible clues of treatment for patients with the infection of COVID-19. Furthermore, the research and development of a new drug need consume a vast of time and resource. Hence, it may be a more appropriate strategy to screen anti-SARS-CoV-2 drug candidates from existing FDA-approved drugs.

In this manuscript, we arrange three VDA datasets including VDA matrix, virus complete genomic sequence similarity matrix, and drug chemical structure similarity matrix. First, virus GAPK similarity and drug GAPK similarity are computed. Second, the final similarity is obtained by integrating biological similarity and GAPK similarity. Third, a bounded nuclear norm regularization model is developed to predict anti-SARS-CoV-2 drug candidates. Finally, molecular docking is applied to measure the binding capabilities between the predicted anti-SARS-CoV-2 drugs and the junction of the spike protein-ACE2 interface. Although datasets used in this work is relatively small, we used three VDA datasets to evaluate the performance of VDA-GBNNR. More importantly, antiviral drugs against COVID-19 screened by the proposed VDA-GBNNR method come together in at least two VDA dataset instead of one dataset.

VDA-GBNNR can obtain the best prediction performance. It may mainly be the following advantages. First, GAPK similarity can effectively depict the association similarity between two viruses (or drugs). Second, the proposed bound nuclear norm regularization model can reduce the overfitting problem. Finally, range constraint makes all the predicted association scores can be within a predefined range.

## Conclusion

In this study, we integrate the heterogeneous virus-drug network and design a VDA prediction model based on bounded nuclear norm regularization to explore potential anti-SARS-CoV-2 drugs. Experimental results show that VDA-GBNNR is an effective VDA identification method. The six FDA-approved drug candidates are found to be potential antiviral drugs against SARS-CoV-2. We hope that the inferred drugs can contribute to the inhibition of COVID-19.

In the future, first, we will integrate various data resource and build larger dataset. Second, we will consider different computational models (Gaur and Chaturvedi, 2019; Liu et al., 2019; Gutiérrez-Cárdenas and Wang, 2021), for example, matrix decomposition (Chen et al., 2018a), bidirectional label propagation (Wang et al., 2019), network distance analysis (Zhang et al., 2021), internal confidence-based collaborative filtering recommendation (Wang et al., 2020b), sparse subspace learning with Laplacian regularization (Chen et al., 2017) to search possible associations between viruses and drugs. Third, we will try to use deep learning methods to predict drugs for COVID-19 (Wang et al., 2017; Alakus and Turkoglu, 2021; Kang et al., 2021). Finally, we will also investigate the relationship between antimicrobial compounds and COVID-19.

## Data Availability Statement

The original contributions presented in the study are included in the article/supplementary material, further inquiries can be directed to the corresponding author/s.

## Author Contributions

JW, LP, and LZ: conceptualization. LP and LZ: funding acquisition and project administration. LP and JW: investigation and writing-review and editing. JW and LZ: methodology. JW and CW: software. JW and LS: molecular docking and validation. JW: writing-original draft. All authors read and approved the final manuscript.

## Conflict of Interest

The authors declare that the research was conducted in the absence of any commercial or financial relationships that could be construed as a potential conflict of interest. The handling editor declared a past co-authorship with one of the author LZ.

## Publisher’s Note

All claims expressed in this article are solely those of the authors and do not necessarily represent those of their affiliated organizations, or those of the publisher, the editors and the reviewers. Any product that may be evaluated in this article, or claim that may be made by its manufacturer, is not guaranteed or endorsed by the publisher.
